# Gadolinium Chloride Rescues Niemann–Pick Type C Liver Damage

**DOI:** 10.3390/ijms19113599

**Published:** 2018-11-14

**Authors:** Andrés D. Klein, Juan Esteban Oyarzún, Cristian Cortez, Silvana Zanlungo

**Affiliations:** 1 Centro de Genética y Genómica, Facultad de Medicina, Clínica Alemana Universidad del Desarrollo, Santiago 7590943, Chile; 2 Departamento de Gastroenterología, Facultad de Medicina, Pontificia Universidad Católica de Chile, Santiago 8331150, Chile; jeoyarzu@uc.cl; 3 Center for Genomic and Bioinformatic, Faculty of Sciences, Universidad Mayor, Santiago 8580745, Chile; Cristian.cortezplaza@umayor.cl

**Keywords:** Niemann–Pick type C disease, gadolinium chloride, liver disease, cholesterol, therapeutic option

## Abstract

Niemann–Pick type C (NPC) disease is a rare neurovisceral cholesterol storage disorder that arises from loss of function mutations in the *NPC1* or *NPC2* genes. Soon after birth, some patients present with an aggressive hepatosplenomegaly and cholestatic signs. Histopathologically, the liver presents with large numbers of foam cells; however, their role in disease pathogenesis has not been explored in depth. Here, we studied the consequences of gadolinium chloride (GdCl_3_) treatment, a well-known Kupffer/foam cell inhibitor, at late stages of NPC liver disease and compared it with NPC1 genetic rescue in hepatocytes in vivo. GdCl_3_ treatment successfully blocked the endocytic capacity of hepatic Kupffer/foam measured by India ink endocytosis, decreased the levels CD68—A marker of Kupffer cells in the liver—and normalized the transaminase levels in serum of NPC mice to a similar extent to those obtained by genetic *Npc1* rescue of liver cells. Gadolinium salts are widely used as magnetic resonance imaging (MRI) contrasts. This study opens the possibility of targeting foam cells with gadolinium or by other means for improving NPC liver disease. Synopsis: Gadolinium chloride can effectively rescue some parameters of liver dysfunction in NPC mice and its potential use in patients should be carefully evaluated.

## 1. Introduction

Niemann–Pick type C (NPC) is a fatal pediatric neurovisceral cholesterol storage disorder caused by loss of function mutations in the *NPC1* or *NPC2* genes. Both genes encode lysosomal proteins involved in the efflux of free cholesterol from lysosomes [1]. As consequence of these mutations, free cholesterol and other lipids accumulate in NPC lysosomes, generating cellular damage. Although several mechanisms have been postulated to explain the pathogenesis of the disease, the signaling pathways that are specifically affected have not yet been fully elucidated.

The estimated frequency of this disease is 1:100,000 live births [2] and affects many organs including the brain, liver, spleen, and lungs [3]. Some patients develop early liver dysfunction, which is particularly relevant for the infantile forms of the disease. Indeed, a recent study [4] reported the clinical features of 10 NPC patients who developed the disease in the newborn period between 1993 and 2015 and concluded that neonatal presentation is a rare form of NPC with exclusive visceral involvement in the newborn period and poor prognosis leading to premature death due to pulmonary complications and liver failure. About 50% of classic NPC patients suffer from neonatal cholestasis, jaundice, and enlarged liver and/or spleen [5,6]. Of these patients, approximately 10% die from liver failure before they reach 6 months of age [7].

We and other researchers have reported progressive inflammation, oxidative stress, apoptosis, and fibrosis in the liver of a murine model of the disease (*Npc1^−/−^* mice) [8,9,10]. From a histopathological perspective, NPC livers show large amounts of CD68-positive foam cells; however, their role in disease pathogenesis has been poorly characterized. It has been proposed that accumulation of Kupffer cells loaded with lipids would lead to the consequent secretion of inflammatory cytokines, such as TNFα and profibrotic TGF-beta [9,11]. This response, in other diseases with hepatic lesions, leads to the recruitment and activation of hepatic stellate cells (HSC), triggering fibrosis and apoptosis. In this scenario, Kupffer cells would have an essential role in NPC liver pathogenesis. To test this hypothesis, we treated NPC mice with gadolinium chloride (GdCl_3_), a well-known Kupffer/foam cell inhibitor [12].

GdlCl_3_ has been widely used to examine the relevance of Kupffer cells in the pathogenesis of several liver diseases. In fact, it has been used in different models, including drug-induced damage, arsenic toxicity, diet-induced hepatic steatosis, and insulin resistance and ischemia–reperfusion [13,14,15,16]. In these and other studies, it was reported that GdCl_3_ prevents or diminishes liver damage. More recently, Yang et al. demonstrated that GdCl_3_ attenuated liver inflammation and fibrosis in two liver injury models (methionine-choline deficient and high fat (MCDHF) and carbon tetrachloride (CCl_4_)) [17]. On the other hand, Kupffer cell inhibition can induce detrimental effects. It has been observed that treatment with GdCl_3_ decreases hepatic regeneration after partial liver transplantation [18]. Similarly, the work of Zhu et al. shows that Kupffer cell depletion by gadolinium chloride aggravates liver injury after brain death in rats [19]. 

Besides inhibiting the endocytosis of Kupffer cells, GdCl_3_ depletes the M1 subpopulation of macrophages. This subset of cells corresponds to proinflammatory macrophages activated by LPS and Th1 proinflammatory cytokines, such as IFNγ, TNFα, and IL-1β. GdCl_3_ would not affect the M2 subset, macrophages that participate in resolution of inflammation, and comprises various forms of nonclassically activated macrophages originating from exposure to different stimuli, such as the Th2 cytokines IL-4, IL-13, IL-10, and TGFβ [20].

Gadolinium salts are widely used in clinics as MRI contrasts [21]. These salts have been used for studying the in vivo function of Kupffer cells, since circulating monocytes and other macrophages are less vulnerable to them [22]. In the present study, we evaluated the effects of GdCl_3_ on NPC liver disease and we compared them with a genetic rescue of NPC1 protein in hepatocytes in vivo, which was previously shown to be sufficient to decrease liver pathology [23].

## 2. Results

**Figure 1:**
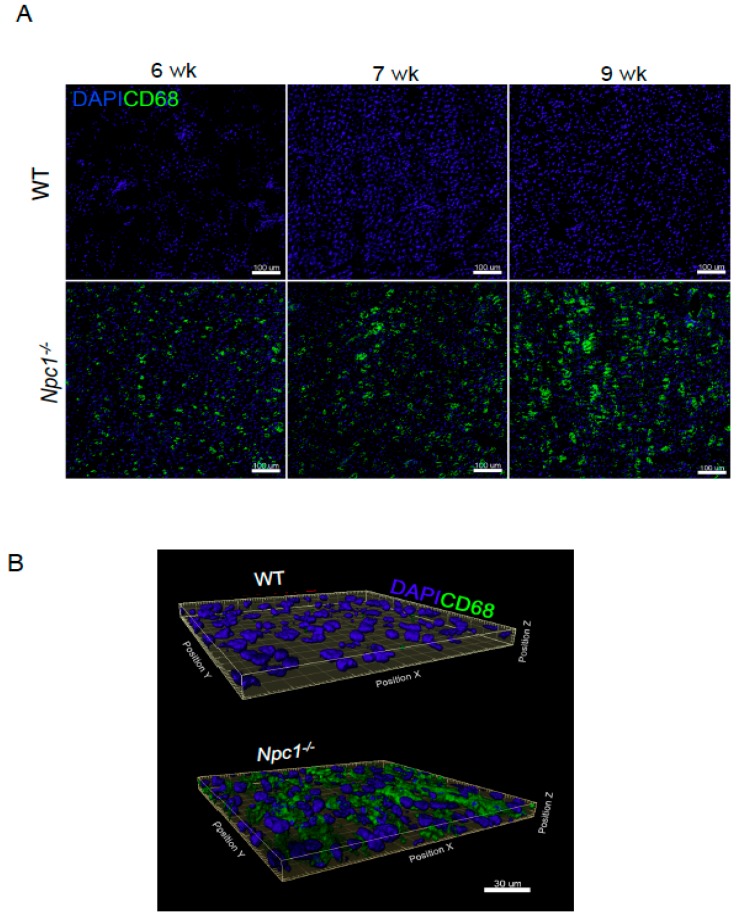
Progressive increase of hepatic CD68 positive cells in Niemann–Pick type C (NPC) mice. (**A**) CD68 staining of livers from 6-, 7-, and 9-week-old (wk) wild-type (WT) and *Npc1^−/−^* mice. Scale bars are 100 µm. (**B**) Three-dimensional reconstructions of liver sections from 9-week-old WT and *Npc1^−/−^* mice.

To study the kinetics of hepatic macrophage infiltration, we analyzed the levels of CD68 staining by immunofluorescence. No foam cells were found in wild-type (WT) mice, whereas increased staining was observed in NPC (*Npc1^−/−^*) tissues. Moreover, the levels of the staining positively correlated with the age of the *Npc1^−/−^* mice (Figure 1A), suggesting that foam cells might play a role in liver pathogenesis. To further characterize the extent of foam cell infiltration at late stages of disease progression, we performed a three-dimensional reconstruction (z-stacks) of representative liver sections from 9-week-old animals. *Npc1^−/−^* livers showed many round-shape CD68 foam cells compared to wild-type (WT) tissues (Figure 1B). 

**Figure 2:**
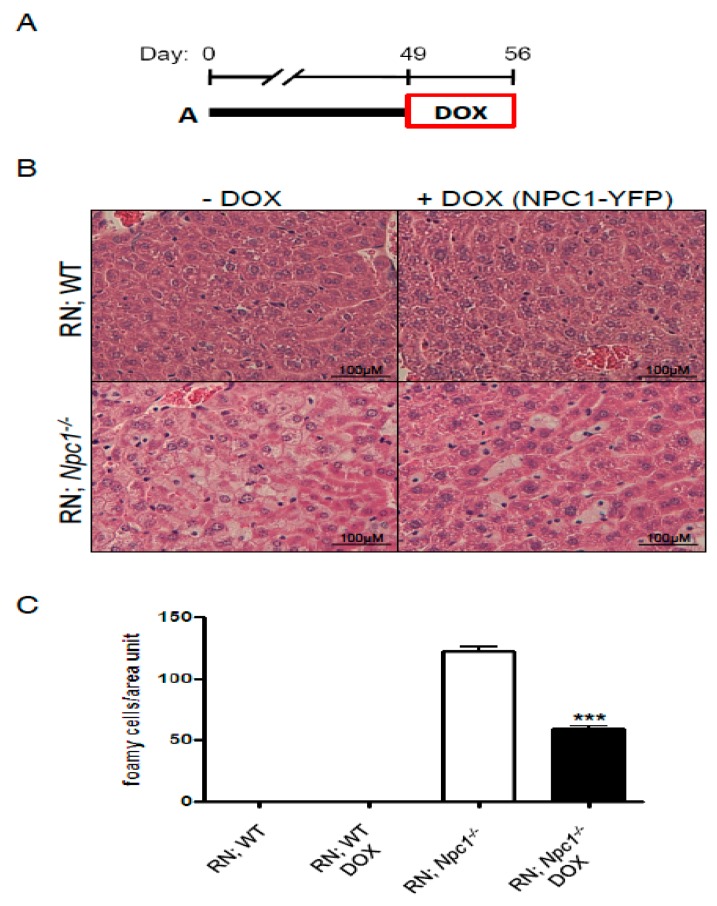
Foam-cell-associated NPC liver pathology is corrected by genetic NPC1 rescue. (**A**) Timeline of the duration and age of doxycycline (DOX) treatment in mice. (**B**) Expression of the NPC1-YFP transgene, driven by Rosa-rtTA in ROSA26-rtTA-M2 and TRE-Npc1-YFP (R; N), WT, or *Npc1^−/−^* mice, was induced with DOX on day 49. After 1 week of treatment, animals were sacrificed and livers were processed for histological analysis by Hematoxylin and eosin staining. Scale bars are 100 µm. (**C**) Quantification of the number of foam cells in each condition. *** *p* < 0.0001.

Next, we analyzed the extent of reversibility of foam cell numbers after rescuing hepatic NPC1 by genetic means. For this purpose, we used a previously developed genetic tool to express a tagged-NPC1 protein in hepatocytes in vivo based on the tet system [23,24]. Upon doxycycline (DOX) administration to *Npc1^−/−^* transgenic mice for ROSA26-rtTA-M2 and TRE-Npc1-YFP (abbreviated R; N), NPC1-YFP was expressed. Hepatocyte correction was sufficient to rescue NPC liver damage [23]. We then continued characterizing this effect in terms of the number of foam cells. We allowed the disease to progress for 49 days, when mice already show liver disease, and we fed them with DOX for 1 week (Figure 2A). Tissues were stained with hematoxylin and eosin at postnatal day 56 (Figure 2B). As previously shown for the BALB/c background, wild-type animals did not show the presence foam cells. Livers from nonrescued *Npc1^−/−^* mice showed a large number of foam cells/area unit, which was significantly reduced upon DOX treatments (Figure 2C). Therefore, we hypothesized that inhibition of foam cells may have a positive impact on NPC liver disease.

**Figure 3:**
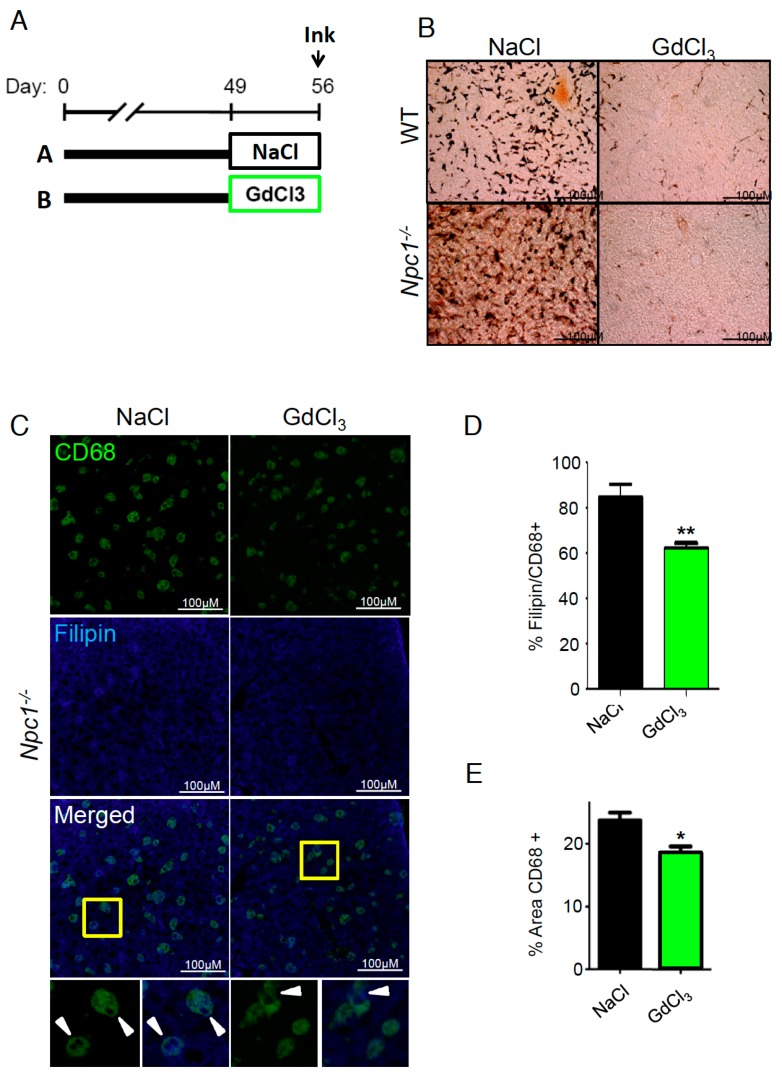
GdCl_3_ treatment decreases Kupffer/foam cells endocytosis. (**A**) Timeline of the duration and age of GdCl_3_ treatment in mice. The time of India ink injection is indicated (postnatal day (P)56). As a control, animals were treated with a saline solution (NaCl). (**B**) India ink deposits were visualized with light microcopy. Scale bars are 100 µm. (**C**) CD68 and filipin staining in GdCl_3_- and NaCl-treated *Npc1^−/−^* mice. Scale bars are 100 µm. Bottom images show merged CD68 and filipin staining and the areas marked with yellow squares were magnified below. The arrowheads show cells positive for CD68 and filipin. (**D**) Quantification of % filipin/CD68 positive cells. (**E**) Quantification of the area covered by CD68 cells.

If activation of foam cell activity plays a detrimental role in NPC liver disease, their inhibition should exert beneficial effects. To test the technical feasibility of inhibiting hepatic Kupffer/foam cell endocytosis, we treated animals with GdCl_3_ from postnatal day (P)49–P56 followed by one India ink injection (Figure 3A). We decided to treat *Npc1^−/−^* animals starting from P49 because at this stage, they already showed extensive liver damage, including increased alanine transaminase (ALT) and inflammation [10,23]. We chose the therapeutic dose of 10 mg/kg of GdCl_3_ because it had been successfully used before in models presenting hepatic buildup of lipids and in other animal models with liver extensive damage [16,25,26]. The saline-treated animals showed high levels of India ink deposits in the liver with a distribution pattern consistent with Kupffer cell morphology in WT animals and foam cells in *Npc1^−/−^* mice. These deposits were difficult to find in animals pretreated with GdCl_3_ (Figure 3B), indicating that gadolinium effectively blocks the endocytic properties of these cells under WT conditions and also in the context of NPC disease. 

**Figure 4:**
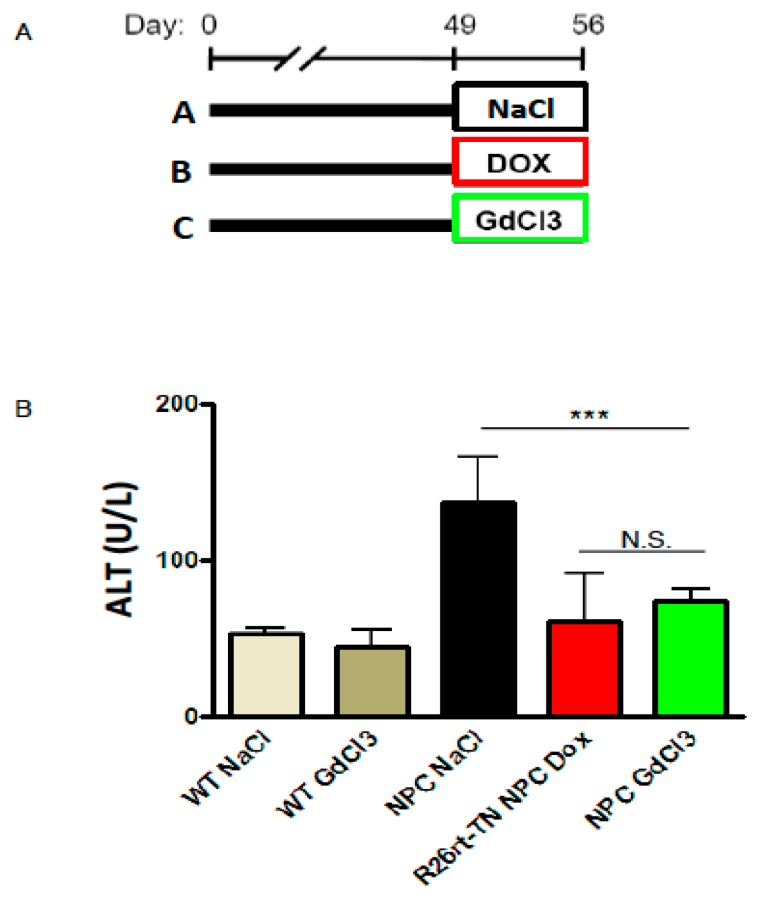
Foam-cell-associated NPC liver pathology is improved by GdCl_3_ treatment. (**A**) Timeline of the duration and age of different treatments in mice. (**B**) Alanine transaminase (ALT) levels in the different groups. Data are presented as means ± SD. (*n* = 3–5 animals/group, two-way ANOVA, and Tukey’s multiple comparison post-test). *** *p* < 0.0001.

To characterize the effects of blocking NPC foam cell endocytosis and their cholesterol buildup, we stained liver sections with CD68 and filipin. GdCl_3_-treated livers have a decreased number of CD68 cells and CD68 cells positive for filipin. This effect can be visualized in the merged images (Figure 3C) and it was also quantified (Figure 3D,E). GdCl_3_ not only blocks endocytosis but also depletes the M1 subpopulation of macrophages in WT animals [22]. To test the effects of GdCl_3_ in an NPC context, we used CD68 staining as a general marker of Kupffer/foam cells. Interestingly, GdCl_3_ treatment from P49 to P56 reduced the area covered by CD68 cells (Figure 3E). Finally, to determine the effect of GdCl_3_ treatment on liver disease, we measured the levels of the ALT enzyme in plasma (Figure 4A). Importantly, we found that GdCl_3_ significantly reduced ALT levels in NPC mice to levels very similar to those produced by genetic rescue of NPC1 (Figure 4B).

Together these results show that GdCl_3_ can effectively rescue Niemann–Pick type C liver ALT increase in mice.

## 3. Discussion

Although neuronal damage is the major cause of death in NPC, most patients have considerable liver damage. In fact, several studies indicate that between 45% and 65% of NPC patients develop liver disorders, including cholestasis, prolonged jaundice, and hepatomegaly [5,6]. The engagement of immune pathways in NPC pathogenesis has shown disparate results [27,28,29,30,31]. In this study, we demonstrated that GdCl_3_ decreased the endocytic capacity of Kupffer/foam cells and normalized transaminase levels in serum of NPC mice to a similar extent to those obtained by NPC1 genetic rescue of liver cells. 

Gadolinium treatment has shown beneficial effects in several models of liver diseases, including alcoholic liver disease [32], nonalcoholic fatty liver [13], liver failure following acetaminophen intoxication [33], ischemia–reperfusion injury [14], amiodarone [16], and others. Amiodarone-induced liver damage is particularly interesting for our work since it induces a Niemann–Pick-type-C-like phenotype in cells [34]. In animal models, 10 mg/kg of GdCl_3_ has been shown to be safe. However, 50 mg/kg induces kidney toxicity [35]. In humans, there is still debate about the GdCl_3_ safety range. To date, the main reported adverse symptom is nephrogenic systemic fibrosis in patients with renal disease already present. These patients do not receive GdCl_3_ but other Gd-based contrasts. However, it should be kept in mind that GdCl_3_ can be accumulated in the human body, inducing kidney toxicity and potentially the dysfunction of other organs by unknown mechanisms [36]. Toxicity depends on the molecular stability of the drug, which varies together with the pharmacokinetic properties of each compound and also depends on the individual characteristics of the subject [37]. In children, the rate of Gd-induced acute adverse nephropathy is lower than in adults [38]. However, recent findings indicate that Gd can accumulate in other tissues, including the brain, in patients with normal renal function. These data added renewed concerns regarding Gd long-term toxicity [39].

The mechanisms by which GdCl_3_ exerts its beneficial effects on livers of NPC mice are not entirely clear. However, they are not mediated by NPC1 (since GdCl_3_ can rescue a NPC1 deficient mice) but rather by modulating signaling pathways triggered by uncontrolled lipid uptake by macrophages. It is very likely that macrophages from genetically rescued livers secrete lower levels of inflammatory chemokines and cytokines because they have been uptaking lower amounts of lipids. A potential downstream effector of NPC macrophages that could be blocked by GdCl_3_ is TNFα, which has been previously shown to play a role in hepatic NPC pathophysiology [9]. Therefore, GdCl_3_ effects could be directly related to the inactivation of Kupffer cells in response to the accumulation of cholesterol and/or to a reduction in the secretion of cytokines such as TNFα. Indeed, a large number of studies show that Gd^3+^ is a selective Kupffer cell inhibitor that lowers Kupffer cell number and phagocytic activity [40,41,42,43]. Moreover, Vincent et al. (2010) showed that antibodies against TNFα decreased some parameters of liver damage in NPC mice [44]. Another possibility is that gadolinium could correct calcium homeostasis or sphingosine accumulation, which are disease hallmarks [45,46]. The increase in lysosomal sphingosine levels would increase the release of calcium from NPC lysosomes, emptying their stores [47]. Release of lysosomal calcium is required for lysosome trafficking and fusion with other cellular compartments, such as autophagosomes and the plasma membrane [48]. Increasing the expression or activity of the Mucolipin 1 or TRPML1 lysosomal ion channel is sufficient to correct the trafficking defects and reduce lysosome storage and cholesterol accumulation in NPC cells [49]. Interestingly, Gd^3+^ is a lanthanide that exhibits properties similar to that of calcium in biological systems, presumably due to its comparable ionic radius [50], and is detected in lysosomes of hepatocytes and Kupffer cells [51]. Considering these antecedents, gadolinium could potentially activate TRPML1. According to this idea, we found that gadolinium slightly decreased filipin staining in CD68 positive cells in the treated mice. These results suggest that the mechanism by which GdCl_3_ exerts its beneficial effects is mediated through the inhibition of foam cell endocytosis rather than other cell types, such as hepatocytes. However, we cannot discard direct effects of GdCl_3_ on hepatocytes. To our knowledge, there are few reports showing protective effects of GdCl_3_ treatments in isolated hepatocytes. It was shown that Gd^3+^ can reduce the levels of cytochrome P450 in hepatocytes [52], which in turn can reduce oxidative damage. Oxidative damage is an important feature in NPC; therefore, Gd^3+^ can contribute to the beneficial effects observed in vivo.

In addition, the effects of GdCl_3_ could be related to its activity as an inducer of the macrophage M1 subpopulation depletion [22]. Interestingly, we have previously shown that treatment with clodronate, which mainly depletes M2 macrophages [53], enhanced NPC liver damage [23]. Further studies are required to determine the mechanisms by which gadolinium improves liver disease in NPC mice.

Recent studies show that it is possible to improve liver disease in NPC by trying combined therapies that include cyclodextrin to decrease the accumulation of cholesterol. Alam et al. (2016) obtained outstanding results, both in viscera and CNS, treating an NPC mouse model with the pan-HDAC inhibitor (HDACi) vorinostat, 2-hydroxypropyl-β-cyclodextrin (HPBCD), and polyethylene glycol (PEG) [54]. Ebner et al. (2018) tried a combination of HPβCD, miglustat, and allopregnanolone, obtaining a decrease in hepatic lipids and an amelioration of mouse NPC liver disease symptoms [55]. In this sense, gadolinium can be visualized as an alternative for a combination therapy along with other drugs such as cyclodextrin, capable of lowering the cholesterol burden, specifically directed to improve liver function in NPC disease. 

In summary, our work shows that gadolinium inhibits Kupffer cell activity and normalizes ALT levels in serum of NPC mice. Our research opens the possibility of targeting foam cells with gadolinium or by other means for improving NPC liver disease.

## 4. Materials and Methods

### 4.1. Mice

BALB/c *Npc1^+/−^* mice, originally purchased from Jackson Laboratory (Bar Harbor, ME), were bred to generate our own colony at Pontificia Universidad Católica de Chile (PUC). Genotypes (*Npc1^+/+^;* WT and *Npc1^−/−^*; NPC) were identified as described previously [56]. In addition, we used *Npc1^−/−^* mice in the FVB background, which develop a disease indistinguishable from the animals in the BALB/c background [23,24]. Transgenic *ROSA26-rtTA-M2*; *TRE-Npc1-YFP*, *Npc1^−/−^* mice for liver rescue were in the FVB genetic background. The breeding strategy to generate Npc1^−/−^ transgenic mice for ROSA26-rtTA-M2 and TRE-Npc1-YFP (R;N) and genotyping protocols have been previously reported [24]. *Npc1^−/−^* mice in the FVB background were used for GdCl_3_ treatments to compare the results with the genetic rescue experiments. The experiments performed with animals in the FVB background were done at Stanford University School of Medicine and those performed in the BALB/c background were executed at PUC. Protocols were performed according to accepted criteria for the humane care of experimental animals and were approved by the institutional review board of both institutions (Stanford Administrative Panel on Laboratory Animal Care (APLAC) protocol #10412 and Scientific Ethics Committee for the Care of Animals and the Environment, Pontificia Universidad Católica de Chile #160321008 (exCEBA 14-038), approved on 6 November 2014). 

### 4.2. Treatments

All mice had free access to water and chow diet (<0.02% cholesterol; Prolab RMH 3000, PMI Feeds Inc., St. Louis, MO, USA) until they were used for the studies. Doxycycline (Sigma, Kawasaki City, Japan) was administered in the drinking water at 2 mg/mL in 5% sucrose, every 3 days, from P49–P56. GdCl_3_ (Sigma, Kawasaki City, Japan) was injected at 10 mg/Kg i.v. (dissolved in NaCl 0.9%) on days 49 and 53, and tissues and the liver enzymes were processed on day 56. India ink injections were administered i.v. 30 min before sacrificing the animals as previously described [57].

### 4.3. Liver and Blood Sampling

Mice were anesthetized by intraperitoneal injection of ketamine (80–100 mg/kg) and xylazine (5–10 mg/kg). After sacrificing the animals, blood was extracted from the inferior vena cava. Blood was centrifuged at 4000 rpm for 20 min at 4 °C to recover the plasma, which was subsequently stored at −20 °C. 

### 4.4. Liver ALT

ALT plasma level analysis was performed in-house at the Stanford University Veterinary Service.

### 4.5. Histology

For the BALB/c *Npc1^+/+^* and *Npc1^−/−^* mice, fresh tissues were frozen in liquid nitrogen. They were sectioned with a cryostat in 8-μm slices and then mounted on slides. They were fixed for 30 min in 4% paraformaldehyde. The cryosections were washed three times with 0.05% PBS-tween. Then, they were permeabilized with 0.1% Triton, blocked at room temperature for 1 h in 4% BSA, and incubated with the mouse anti-CD68 antibody (1:1000; Serotec, Kidlington, UK). CD68 was detected using an anti-rat donkey antibody conjugated with Cy3 (1:500; Jackson Immuno Research Laboratories, West Grove, PA, USA). Confocal images of immunostained tissue were acquired with a Leica TCS SP8 laser-scanning microscope (Leica, Wetzlar, Germany). The series of images obtained from confocal z-stacks were processed and analyzed using Leica LAS AF (Leica, 2012, Wetzlar, Germany). In addition, 3D reconstructions of z-stack image data obtained from wild-type or *Npc1^−/−^* slices acquired in confocal microscopy were processed and analyzed using the Imaris software (Bitplane, Zürich, Switzerland).

For the animals in the FVB background, procedures were performed as previously described [23,58]. Briefly, free-floating liver sections were blocked for 1 h with 2% BSA/0.2% Triton X-100 in PBS and incubated overnight with a rat anti-CD68 antibody (1:1000; Serotec, Kidlington, UK). CD68 was detected using a Cy3-conjugated donkey anti-rat antibody (1:200; Jackson Immuno Research Laboratories, West Grove, PA, USA). Filipin was obtained from Sigma. The percent area of tissue positive for CD68 immunofluorescence was measured using the ImageJ 1.50i software (NIH, Bethesda, MD, USA) as previously described [24]. Hematoxylin and eosin staining was performed by the pathological anatomy facility at Stanford University. To detect India ink deposits, livers were removed 30 min postinjection, fixed in 4% paraformaldehyde overnight, and sectioned with a Vibratome into 40-µm slices. Tissues were mounted on a cover slide and observed directly with a Zeiss Axioplan2 microscope equipped with an AxioCam HRc CCD camera. Confocal images were obtained with a Leica TCS SP2 laser-scanning microscope. Quantification of the number of foam cells in the genetically rescued mice and the filipin-positive CD68 cells were performed manually. Quantification of CD68 fluorescence was performed as previously described [24]. Briefly, CD68 staining was converted to binary data by manual thresholding using ImageJ [59], so that the space occupied by the resulting black image approximated the area of fluorescence. The percentage area occupied by black pixels was then measured and the SEM reported. 

### 4.6. Statistical Analyses

Statistical analyses were performed using GraphPad Prism 6 (Graphpad Software Inc., San Diego, CA, USA). ANOVA followed by Tukey’s multiple comparison test were used for statistical comparisons between the groups. Data in graphs are shown as mean ± SEM.
